# Muscle and epidermal contributions of the structural protein β-spectrin promote hypergravity-induced motor neuron axon defects in *C. elegans*

**DOI:** 10.1038/s41598-020-78414-y

**Published:** 2020-12-03

**Authors:** Saraswathi S. Kalichamy, Alfredo V. Alcantara, Ban-Seok Kim, Junsoo Park, Kyoung-hye Yoon, Jin I. Lee

**Affiliations:** 1 Division of Biological Science and Technology, College of Science and Technology, Yonsei University, Mirae Campus 304, 1 Yonseidae-gil, Wonju, Gangwon-do 26493 South Korea; 2 Department of Physiology, Mitohormesis Research Center, Yonsei University Wonju College of Medicine, Wonju, Gangwon-do 26426 South Korea

**Keywords:** Axon and dendritic guidance, Development, Caenorhabditis elegans, Cell adhesion, Developmental biology, Genetics

## Abstract

Biology is adapted to Earth’s gravity force, and the long-term effects of varying gravity on the development of animals is unclear. Previously, we reported that high gravity, called hypergravity, increases defects in the development of motor neuron axons in the nematode *Caenorhabditis elegans*. Here, we show that a mutation in the *unc-70* gene that encodes the cytoskeletal β-spectrin protein suppresses hypergravity-induced axon defects. UNC-70 expression is required in both muscle and epidermis to promote the axon defects in high gravity. We reveal that the location of axon defects is correlated to the size of the muscle cell that the axon traverses. We also show that mutations that compromise key proteins of hemidesmosomal structures suppress hypergravity-induced axon defects. These hemidesmosomal structures play a crucial role in coupling mechanical force between the muscle, epidermis and the external cuticle. We speculate a model in which the rigid organization of muscle, epidermal and cuticular layers under high gravity pressure compresses the narrow axon migration pathways in the extracellular matrix hindering proper axon pathfinding of motor neurons.

## Introduction

Organisms and biological systems constantly adapt to and evolve with their changing environments. However, earth’s gravity is a static environmental parameter that remains unchanged through time. Thus, biological processes on earth are not adapted to conditions of space gravity, called microgravity, or high gravity, called hypergravity. Studying the effects of gravity on health and basic biology is a challenge due to the difficulty in altering gravity force, but a clearer understanding of how microgravity and hypergravity can alter the body will be necessary as space travel and habitation become more frequent in the near future. True microgravity conditions can be only experienced in spaceflight. Observations of astronauts, as well as biological experiments during spaceflight have shown that muscle and bone mass decrease in microgravity conditions^1,2^. In human twin studies, long-term space habitation resulted in specific gene expression changes that in some cases appear to be permanent^3^. Studying the effects of microgravity on neurons and the nervous system development is even more challenging. Studies in microgravity have shown small differences in cerebellar cortex and vestibular system neuron structure in rats^4,5^. However larger scale nervous systems experiments have not occurred, and questions of gravity’s effect on neuron development remain obscure.

The effects of hypergravity on some aspects of nervous system and brain structure has also been evaluated. In vertebrates including fish, frogs and rats, hypergravity induces a reduction and slowing of brain development^6–9^, and a reduction in the number of Purkinje cells in the cerebellum^10^. In addition, hypergravity exposure in embryonic rats altered the development of the axons of vestibular neurons^11^. How hypergravity alters genetic and cellular environments to cause such changes in the nervous system is not known. Studies suggest that changes in the expression levels of proteins involved with cell–cell interactions such as CD15 and NCAM-L1 may be correlated with hypergravity-induced changes in the brain^9,12^. However, to determine causality of hypergravity-induced changes in the nervous system, genetic experiments should be conducted.

The nematode *Caenorhabditis elegans* has been a valuable tool to study the effects of altered gravity at the genetic level. Studies of *C. elegans* in space have verified that microgravity lowers the expression of muscle development transcription factors to decrease muscle development^13^, alters TGF-β expression to decrease growth of body length^14,15^ and alters fat-related genes and results in decreased accumulation of fat^15^. 100G hypergravity treatment in *C. elegans* does not alter overall muscle integrity but causes nuclear accumulation of the DAF-16 FOXO transcription factor, a conserved stress-response protein^16^. In addition, worms exposed to hypergravity for 12 h in liquid culture had slightly delayed adaptive locomotory responses after treatment^17^.

The DD/VD GABAergic motor neurons control forward and backward movement in *C. elegans*^18,19^. DD neuron axon outgrowth begins in the embryonic stage from cell bodies on the ventral side of the animal and cross the body circumferentially along the body wall to the dorsal side^20,21^. VD neuron axon outgrowth occurs similarly during the L1 larval stage to complete the development of the 19 DD/VD motor neurons. Previously, we showed that hypergravity exposure of at least 10G increases defects in axon development in the DD/VD motor neurons in *C. elegans*^22^. Hypergravity during the L1 larval stage before the VD axons are even born was sufficient to cause an increase in defects, and the increase was consistently observed in all DD/VD axons.

We sought to identify the root cause of hypergravity’s effect on DD/VD axon development. Here, we show that a mutant of *unc-70* that encodes the cytoskeletal protein UNC-70 β-spectrin suppresses hypergravity-induced axon defects. Although β-spectrin protein is known to play roles in the structure of both neurons and muscle, and we find that muscle size is correlated with DD/VD axon defects, our results show that UNC-70 expression in both the epidermis and muscle together promotes hypergravity-induced axon defects. This is supported by genetic analysis of molecules expressed in muscle, ECM, epidermis and the cuticle. Our results promote a model in which proteins that support the proper layered structure of muscle, epidermis and cuticle can become a hindrance in a high gravity force environment.

## Materials and methods

### *C. elegans* culture and strains

*C. elegans* were grown on Nematode Growth Medium (NGM) plates seeded with *E.coli* OP50 and maintained at 20 °C as described^23,24^. Strains used in the experiments were obtained from the CGC and are listed: N2 Bristol strain, LG I: *vab-10(e698)*, *erm-1(tm677)*, *unc-54(s74).* LG II*: vab-19(e1036)*, *unc-52(e1421)*, *rol-6(su1006)*, *juIs76* [*Punc-25::gfp*]. LG IV: *unc-129(ev557)*. LG V: *sma-1(ru18)*, *unc-70(e524).* LG X: *mec-4(e1334)*, *mig-15(rh148)* and *lon-2(n1630)*. For regular genetic maintenance, the *juIs76* strain was outcrossed with N2. In order to visualize the DD/VD axon development in the mutant strains above, we crossed the *juIs76* [*Punc-25::gfp*] strain into the background of single mutants following conventional crossing and screening methods.

### Hypergravity exposure

Hypergravity cultivation chamber was prepared as previously described^22^. For hypergravity experiment, eggs were collected from gravid adult animals by bleaching using standard procedures^25^ and then transferred into the cultivation tube around 100–150 in number. Developing embryos were exposed to 100G hypergravity by setting a temperature-controlled Sorval Legend Micro 17R centrifuge system to 1200 RPM for 60 h at 20 °C similar to previous protocols^22^. Control tubes (1G condition) were maintained in an incubator at 20 °C.

### Axonal defect analysis

After 60 h, worms were collected from the cultivation tube by washing in M9 buffer and were mounted on a 2% agarose pad in 2 µl of 1 M sodium azide to immobilize the worms. An epi-fluorescent microscope (Olympus BX50) was used to observe the DD/VD axons. Axons that do not reach the dorsal nerve cord were considered as a defective axon. In each experiment 100–130 animals were used. For wild-type N2, *juIs76* strain pre-outcrossed and post-outcrossed data were included since differences in axon defects were negligible. Differences between 1 and 100G exposed worms regarding the axonal defects were evaluated by student’s T-test using Excel software (Microsoft, USA). Change in percent defective axons was measured by subtracting percent defects at 100G and 1G for each paired trial, and the differences from each trial were averaged together. Student’s T-test was performed to assess the statistical difference between the change in percent defective axons of N2 and each strain using Excel software (Microsoft, USA).

### Double whole-mount antibody staining of DD/VD axons and muscle cells

Previously described antibody staining of DD/VD axons^26,27^ was modified and performed in the following manner. L4/young adult hermaphrodites for both control and hypergravity-treated samples were mounted and fixed for 1 h at 4 °C in 4% paraformaldehyde/2.5% glutaraldehyde fixative in 1X PBS. To enhance permeation of fixative, samples were frozen in liquid nitrogen and thawed at 70 °C. Before the samples completely melted, we placed them on ice. After fixation, mounted worms were washed thrice in 1X PBS/0.5% Triton X-100 and rocked gently for 18 h at 37 °C in a solution of 5% β-mercaptoethanol, 1% Triton X-100 in 0.1 M Tris–HCl (pH 7.5). The worms were washed four times in 1% Triton X-100/0.1 M Tris–HCl (pH7.5). To quench the autofluorescence caused by glutaraldehyde, the worms were incubated for one hour at 4 °C in a freshly made solution of 1 mg/mL of borate buffer (NaBH_4_, Sigma, 71321) in 1X PBS, with gentle mixing periodically by tapping the bottom of tube. To enhance permeation samples were moved to room temperature and back to ice every 10 min. The worms were then washed twice in PBS buffer. Sample was incubated in 200 µl of 10 mM DTT/ borate buffer (1 mg/ml) for 15 min at room temperature then washed in approximately 200 µl of 1X PBS buffer twice. The sample was incubated in 200 µl 0.3% H2O2/ borate buffer (1 mg/ml) for 15 min at room temperature then washed in approximately 200 µl PBS buffer three times. Blocking was performed with freshly prepared 1% BSA for 1 h at room temperature and then washed in approximately 200 µl PBS buffer three times. For labelling the DD/VD axons, anti-GFP, chicken antibody (Invitrogen, A10262) was used as a primary antibody at 1:1000 dilution in 1X PBS and incubated at room temperature overnight. Alexa Fluor 488 goat anti-rabbit antibody (Invitrogen, 2051237) was used as a secondary antibody at 1:1000 dilution in 1X PBS and incubated for 8 h at room temperature. The samples were washed with 200 µl of 1X PBS. Actin filaments of muscle cells were stained using Phalloidin conjugated Alexa-488 antibody (Invitrogen, A12379) at 1:1000 dilution in 1X PBS and incubated at room temperature for 6 h. Although using different fluorophores in double staining is standard, we decided to use Alexa-488 for both neuron and muscle staining for convenience during microscopic analysis as the two tissue types were easily distinguishable. The samples were washed in PBS once and mounted on 2% agarose pads on a glass slide. A 40× oil objective lens of an epi-fluorescent microscope (Olympus BX20) was used to visualize and photograph the DD/VD axons and muscle cells using CellSens Standard software.

### Measurement of longitudinal area of muscle cells

Images of individual muscle cells were taken using a 40× lens near to the middle of the muscle cells. The outline of muscle cells were traced using the polygon tool in the ImageJ software, and the longitudinal area of the muscle cell was calculated by the software. Student’s T-test was performed to assess the statistical difference between the control and hypergravity samples using Excel software (Microsoft, USA).

To compare axon defects and the dorsal lateral muscle cell each axon traverses, muscle cells were organized into arbitrary regions called R1–R12 (Fig. 4). As there is some animal to animal variability in the muscle that each axon traverses, there is some variability in the muscles that are represented in each region from animal to animal. R1 is defined by muscle 8, (muscle identities as previously defined^20^) which DD2/VD3/VD4 axons pass through. Similarly R2 represents muscle 10 which VD5 traverses, R3 represents muscles 10 or 12, depending on which muscle DD3 traverses, R4 represents muscle 10 or 12 depending on which muscle VD6 traverses, R5 represents muscle 12 or 14 depending on which muscle VD7 traverses, R6 represents muscle 14 which DD4/VD8 traverses, R7 represents muscle 16 which VD9 traverses, R8 represents muscle 18 which DD5/ VD10 traverses, R9 represents muscle 20 which VD11 traverses, R10 represents muscles 20 or 22 depending on which muscle VD12 traverses, R11 represents muscles 20 or 22 depending on which muscle DD6 traverses, and R12 which represent muscles 22 or 24 depending on which muscle VD13 traverses. Longitudinal area of muscle for each region is the average cross-sectional muscle area in a given region for all animals. R^2^ value was determined by regression analysis using Microsoft Excel.

### Measurement of body length

To measure the body length of the worms, gravid adult worms were incubated in bleach solution^25^, and eggs were transferred to a culture plate containing M9 buffer. Eggs hatched within 10–12 h and place onto a standard NGM plate. After 2 h and 18 h the larvae were picked and mounted on a 2% agarose pad, then anaethetisized with 1 M sodium azide. Immobilized worms were photographed with an Olympus BX20 microscope. Body length of the larvae were measured from nose to tip of the tail using ImageJ software^28^.

### Transgenic rescue experiments

Microinjection was performed to express *unc-70* cDNA in *unc-70(e524)*; *juIs76* background. The translational fusion constructs *Ppdi-2::*UNC-70 (epidermal) and *Pmyo-3*::UNC-70 (muscle)^29,30^ were injected into the young adults of *unc-70(e524)*; *juIs76* strain animals using 40 ng/ul of *punc-122::RFP* as a co-injection marker to express UNC-70 in muscle and hypodermis. To investigate whether simultaneous expression of UNC-70 in muscle and hypodermis are required for hypergravity induced axon defects, a 1:1 ratio of *Ppdi-2::*unc-70 and *Pmyo-3*::unc-70 constructs were injected into *unc70(e524);juIs76* strain animals. Stable transgenic lines were obtained at the F2 generation and maintained.

## Results

### Mutations in UNC-70 β-spectrin suppress hypergravity-induced axon defects (HIAD)

**Figure 1:**
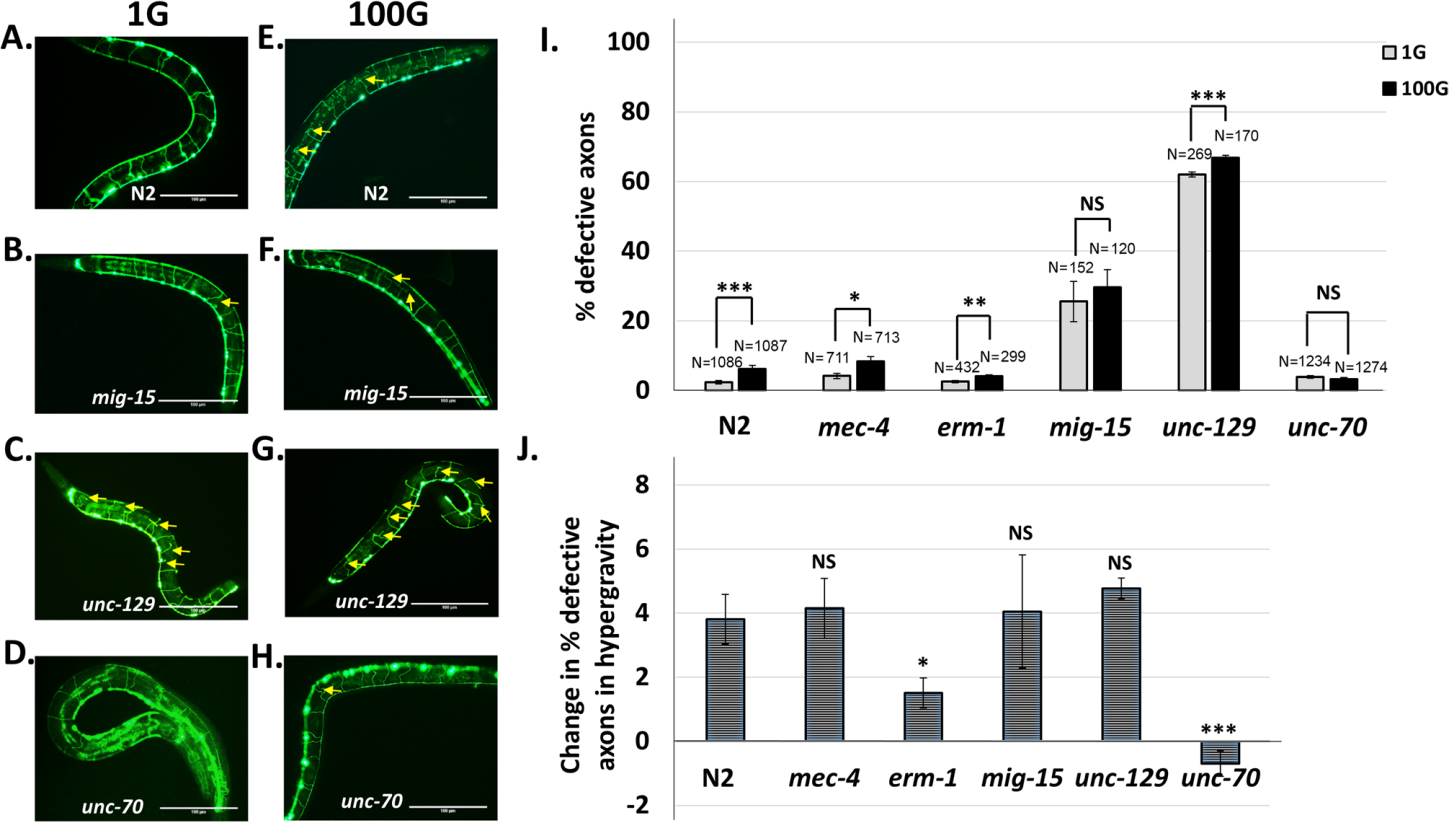
A mutant of the UNC-70 β-spectrin gene suppresses hypergravity-induced axon defects in DD/VD motor neurons. Wild-type (N2) and mutant *C. elegans* strains bearing a GFP marker for DD/VD motor neurons of *C. elegans (juIs76* strain) were exposed to either normal 1G gravity (**A**–**D**) or 100G hypergravity (**E**–**H**) during embryonic and larval development. Yellow arrows indicate axon defects. Scale bar indicates 100 μm. (**I**) Quantification of axon defects in various mutant strains in 1G (grey) or 100G (black) gravity. N = total number of animals analyzed. Statistical significance between 1 and 100G determined by student’s T-test. *p < 0.05, **indicates *p* < 0.01, *** *p* < 0.001, NS indicates no significance. Error bars indicate standard error. (**J**) Increase or decrease in the percent axon defects between 1 and 100G hypergravity. Statistical significance between N2 and each mutant strain determined by student’s T-test. * *p* < 0.05, **p < 0.01, ***p < 0.001, NS indicates no significance. Error bars indicate standard error.

Previously, we showed that hypergravity exposure increases DD/VD motor neuron axon defects^22^. Here, we sought to explain how gravity force can alter the axon development in the worm. It is well established that axon outgrowth and guidance mechanisms allow the axon to reach proper targets. To identify novel factors during early larval development that may contribute to hypergravity-induced axon defects (HIAD), we conducted a candidate genetic screen to find mutations that may exacerbate or suppress HIAD. We measured HIAD by the increase in DD/VD axon defects observed between 1 and 100G in each mutant, and then compared the change in the proportion of axon defects in hypergravity for N2 wild type with each mutant strain (Fig. 1).

To examine whether HIAD is due to neuronal factors, we tested mutants of axon outgrowth and axon guidance. Although most of these mutants already display severe axon defects, we tested HIAD in mutant strains with milder axon phenotypes. *mig-15* encodes serine/threonine kinase protein and *erm-1* encodes an ezrin/radzin/moesin protein, both of which promote growth cone migration of DD/VD axons on encountering longitudinal obstacles^31^. When we subjected mutants of *mig-15(rh148)* and *erm-1(tm677)* to 100G, there was an increase in defects similar to N2 wild type (Fig. 1A, B, E, F, I; Fig S1). *mig-15* mutants started at a higher basal error rate than N2 or *erm-1*, but the increase in error was additive, not synergistic, indicating the mutation does not further exacerbate the axon defect in high gravity (Fig. 1B, F, I). While the increase in axon defects in *erm-1* mutants was significant, the increase observed in *mig-15* mutants was not statistically significant due to a high variation in basal error rate (Fig. 1I; Fig S1). When we compared the increases in axon defects for each trial in *mig-15* mutants and wild type, we found that both strains showed a modest but consistent 4% increase in defects (Fig. 1J). In *erm-1* mutants, the increase in defects was slightly diminished at around 2% (Fig. 1J). To further analyze HIAD, we also calculated the proportion of animals with DD/VD axon defects (Fig. S2). We found that hypergravity significantly increases the portion of *erm-1* mutants with at least one axon defect, but this type of analysis was not useful to assess HIAD in *mig-15* mutants since nearly all the *mig-15* mutants had axon defects at 1G gravity. Taken together, we do not find a large deviation in HIAD between axon outgrowth mutants and the wild-type strain.

*unc-129* encodes a BMP-like TGF-β protein that is secreted by the dorsal body-wall muscle and acts on the dorsal side of the worm during axon migration to increase sensitivity to distant UNC-6/netrin guidance cues^32^. Similar to axon outgrowth mutants, HIAD occurs additively rather than synergistically in 100G in *unc-129(ev557)* mutants (Fig. 1C, G, I, J; Fig S1). The increase in defects in *unc-129* mutants is similar to N2 (Fig. 1J) confirming that the defect is not due to the alteration in DD/VD neuron guidance cues.

A previous study using *C. elegans* showed that hypergravity-dependent nuclear translocation of DAF-16 transcription factor required the *mec-4* gene^16^. The *mec-4* gene encodes the degenerin channel involved in touch sensation^33^. The study suggested that the MEC-4 channel could be involved in gravity sensation. However, *mec-4(e1334)* mutants showed completely normal HIAD similar to wild-type animals (Fig. 1I, J; Fig S1; Fig S2). Overall, disruption of axon outgrowth and migration or lack of touch sensation does not appear to affect HIAD.

We next asked whether HIAD is due to axonal damage. Previous studies have shown that if DD/VD motor neuron axons become damaged, axon regeneration mechanisms can repair the broken axons^34^. Mutants of the *unc-70* gene that encodes the cytoskeletal protein β-spectrin result in fragile DD/VD neuron axons leading to increased damaged axons^29^. We reasoned that if HIAD is the result of axonal damage during hypergravity, we should observe an even higher incidence of defects in *unc-70* mutants, due to the fragile axons. Surprisingly, we found that HIAD was not enhanced but actually suppressed in *unc-70(e524)* mutants (Fig. 1D, H, I, J; Fig S1, Fig S2).

### Muscle and epidermal expression of UNC-70 are important for HIAD

Our results showing that *unc-70* mutants suppress HIAD rather than exacerbate axon defects suggested that UNC-70 was acting in a role separate from its neuronal function. This was consistent with the fact that HIAD requires hypergravity exposure from the embryonic stage up to the end of the L1 larval stage, which is before the VD neurons are even born^22^. In light of this, the fact that hypergravity does not act on multiple neuronal mechanisms such as axon outgrowth, axon guidance and touch sensation was not surprising.

**Figure 2:**
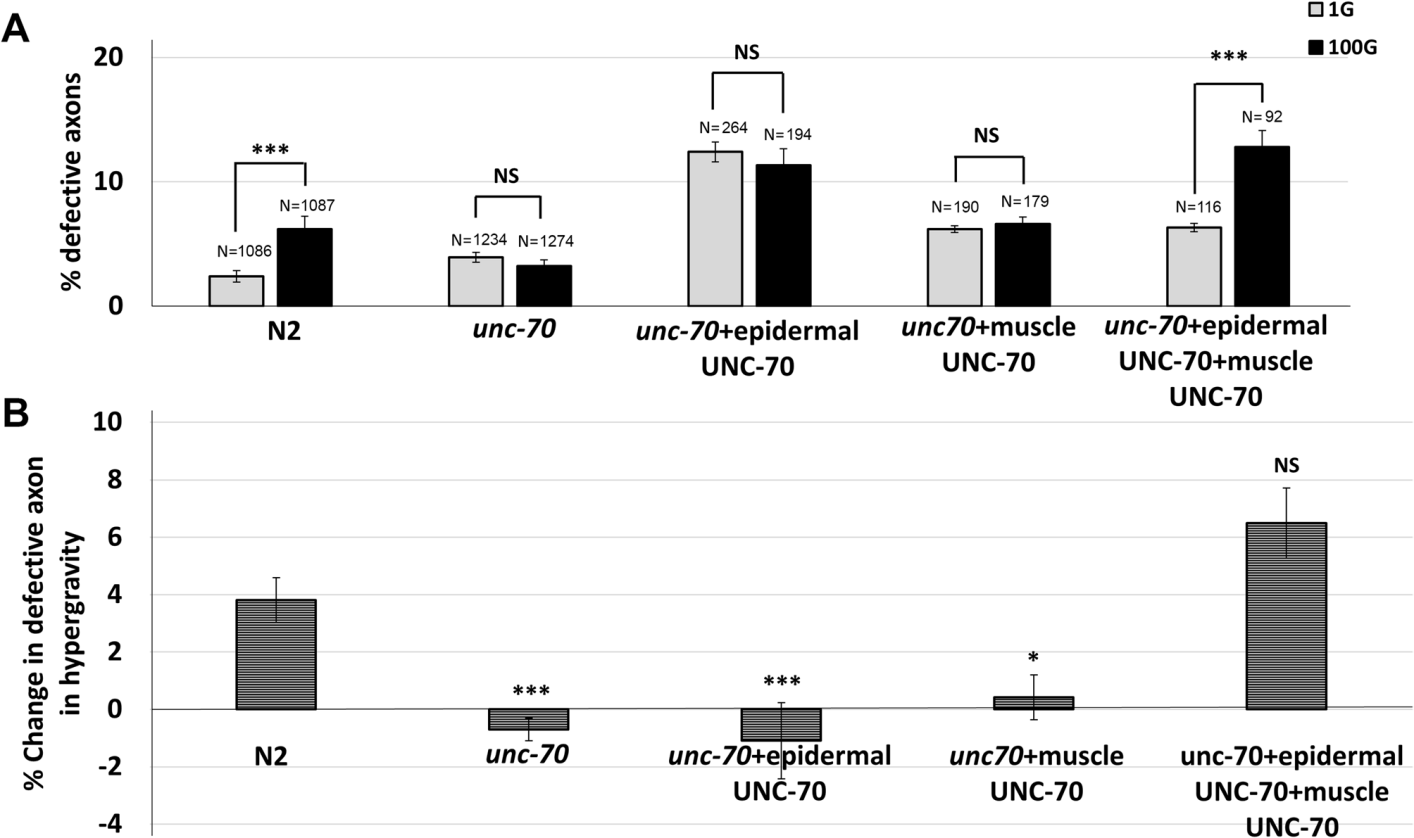
Both epidermal and muscle expression of UNC-70 is required to restore hypergravity-induced axonal defects. (**A**) Transgenic *unc-70; juIs76* animals expressing epidermal UNC-70 (Ppdi-2::unc-70) or muscle UNC-70 (Pmyo-3::unc-70) were exposed to either normal 1G gravity or 100G hypergravity. N = total number of animals analyzed. Statistical significance between 1 and 100G determined by student’s T-test. ***p < 0.001. NS indicates no significance. Error bars indicate standard error. (**B**) Increase or decrease in the percent axon defects between 1 and 100G hypergravity. Statistical significance between N2 and each mutant strain determined by student’s T-test. *p < 0.05, **p < 0.01, ***p < 0.001, NS indicates no significance. Error bars indicate standard error.

Besides neurons, UNC-70 is also expressed in the body-wall muscle and epidermis^35,36^. To address whether UNC-70 functions in these tissues to promote HIAD, we used cell-specific promoters to restore UNC-70 function in the muscle or the epidermis. When *unc-70* was restored in only muscle or only epidermis, we still observed a suppression of hypergravity-induced axon defects (Fig. 2A, B). Thus, muscle or epidermal *unc-70* alone was not sufficient to restore normal HIAD. However, when *unc-70* was restored in both muscle and hypodermis simultaneously, we were able to once again observe hypergravity-induced axon defects similar to N2 (Fig. 2A, B). Thus, both muscle and epidermal expression of UNC-70 is required for HIAD.

### DD/VD neuron axon defects occur at the juncture with the dorsal lateral muscle

How could both the muscle and epidermal tissue be responsible for the axon defects observed in hypergravity? An earlier study provided a clue: the authors of this study showed that during axon development, the VD axon growth cones quickly move circumferentially towards the dorsal side until it reaches the juncture with the lateral-most end of the dorsal muscle^37^. At this point the VD growth cone stalls for 4 h, collapses and re-forms, then slowly navigates through the narrow basal lamina/ECM between the muscle and epidermis towards the dorsal nerve cord^37^. This delay in VD axon migration is thought to be due to (1) the muscle acting as a physical barrier to migration, and (2) the presence of dense fibrous organelles (FOs) in the basal lamina/ECM between the muscle and epidermis. FOs are hemidesmosomal-like structures originating from the epidermal cells that densely line the space between the muscle and epidermis^37–39^. UNC-70 in the epidermis is known to maintain the stability of FO structures that connect muscle with the epidermis^40^. In the muscle cells UNC-70 localizes to membrane attachment sites of the myofilament apparatus and is important for proper muscle structure and organization^35,36^.

**Figure 3:**
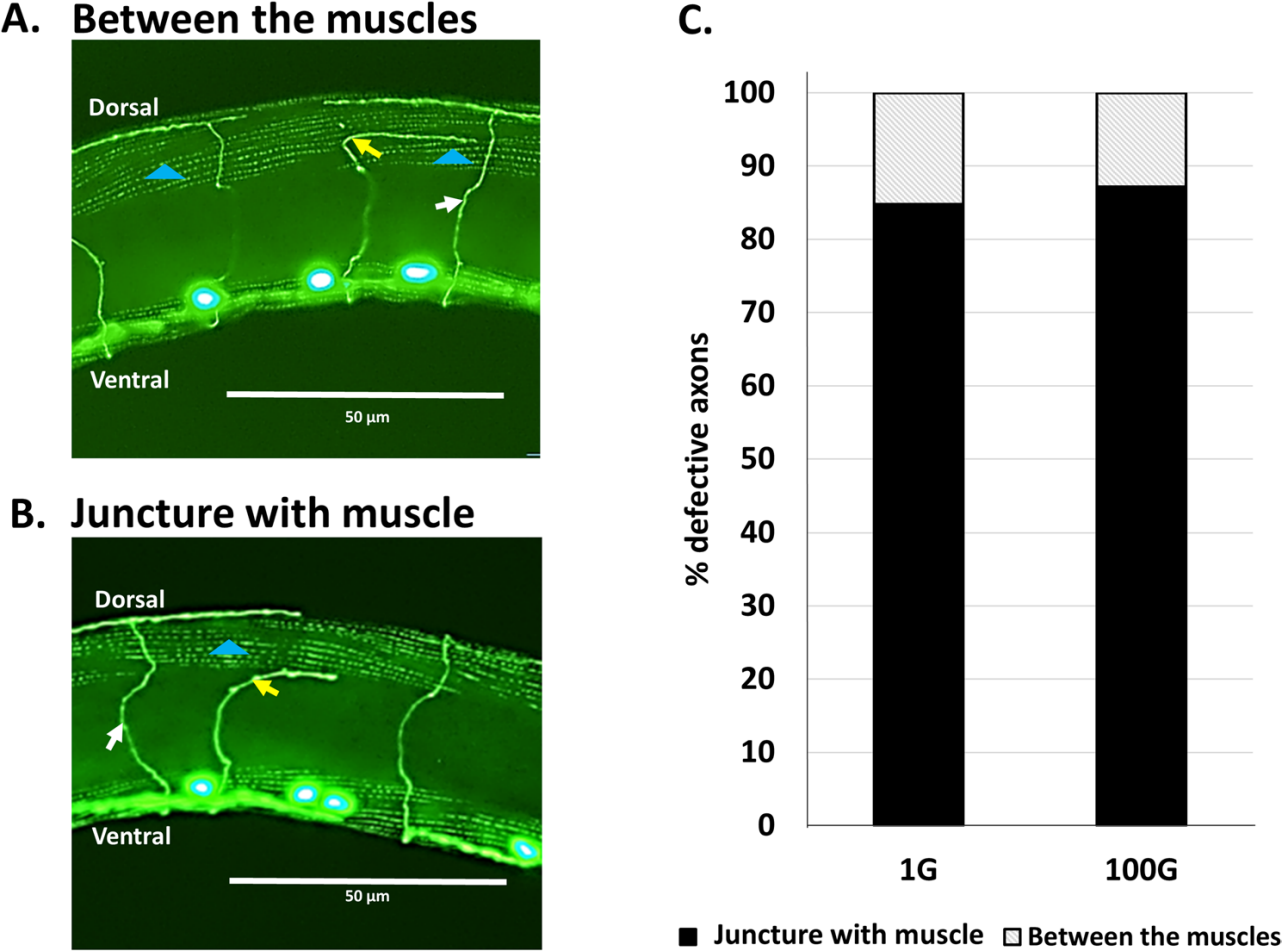
Most axon defects occur at the juncture with the dorsal muscle. (**A**, **B**) *juIs76* strain animals bearing the DD/VD neuron GFP marker were stained with Alexa 488-conjugated phalloidin antibody. Thus, both muscle and axons are stained in green. Axon defects on the dorsal side that occur between the muscles (**A**) or at the juncture with the muscle (**B**). Yellow arrow indicates defective axon. White arrow indicates normal axon. Blue arrowhead indicates dorsal muscle cell. Dorsal and ventral sides indicated. Scale bars indicate 50 μm. (**C**) Most axon defects occur at the juncture with the muscle (black) rather than between the muscles (grey striped).

The results from the above study was in agreement with our previous observation that most of the DD/VD axon errors occurred at the dorsal side of the worm^22^. In addition, almost all axons errors resulted in the axon stopping, branching or turning at a specific point along the circumferential axis of the worm^22^. We wondered if the location of this specific point was at the lateral-most juncture with the dorsal body wall muscle. When we visualized DD/VD axons and the body-wall muscle simultaneously, we immediately noticed a distinct pattern of defects and were able to categorize them into two groups: axon errors occurring at the most lateral juncture with the muscle (Fig. 3A), and errors occurring in the body of the muscle (Fig. 3B). We found that regardless of gravity condition (1G or 100G), most of the defects occur at the most lateral edge of the dorsal muscle rather than in the center of the muscle or more medial dorsal muscle cells (Fig. 3C). This suggests that the dorsal lateral muscle acts as a physical impediment that needs to be overcome for correct axonal pathfinding.

### Incidence of axon defects are correlated with large muscle cells

Previously, we showed that wild-type worms in normal 1G gravity occasionally display DD/VD axon defects^22^. Interestingly, there is a pattern of axon defect distribution in which some of the axons along the anterior–posterior axis consistently show a higher incidence of defects than others. It is unknown, however, what causes the differences in error rates for each DD/VD axon. In light of the evidence that VD axon pathfinding is challenged physically by the narrow basal lamina/ECM space between the muscle and epidermis^37^, we wondered whether there was a relationship between incidence of defects for a given axon and the size of the muscle cell that each axon needs to traverse on the dorsal side.

**Figure 4:**
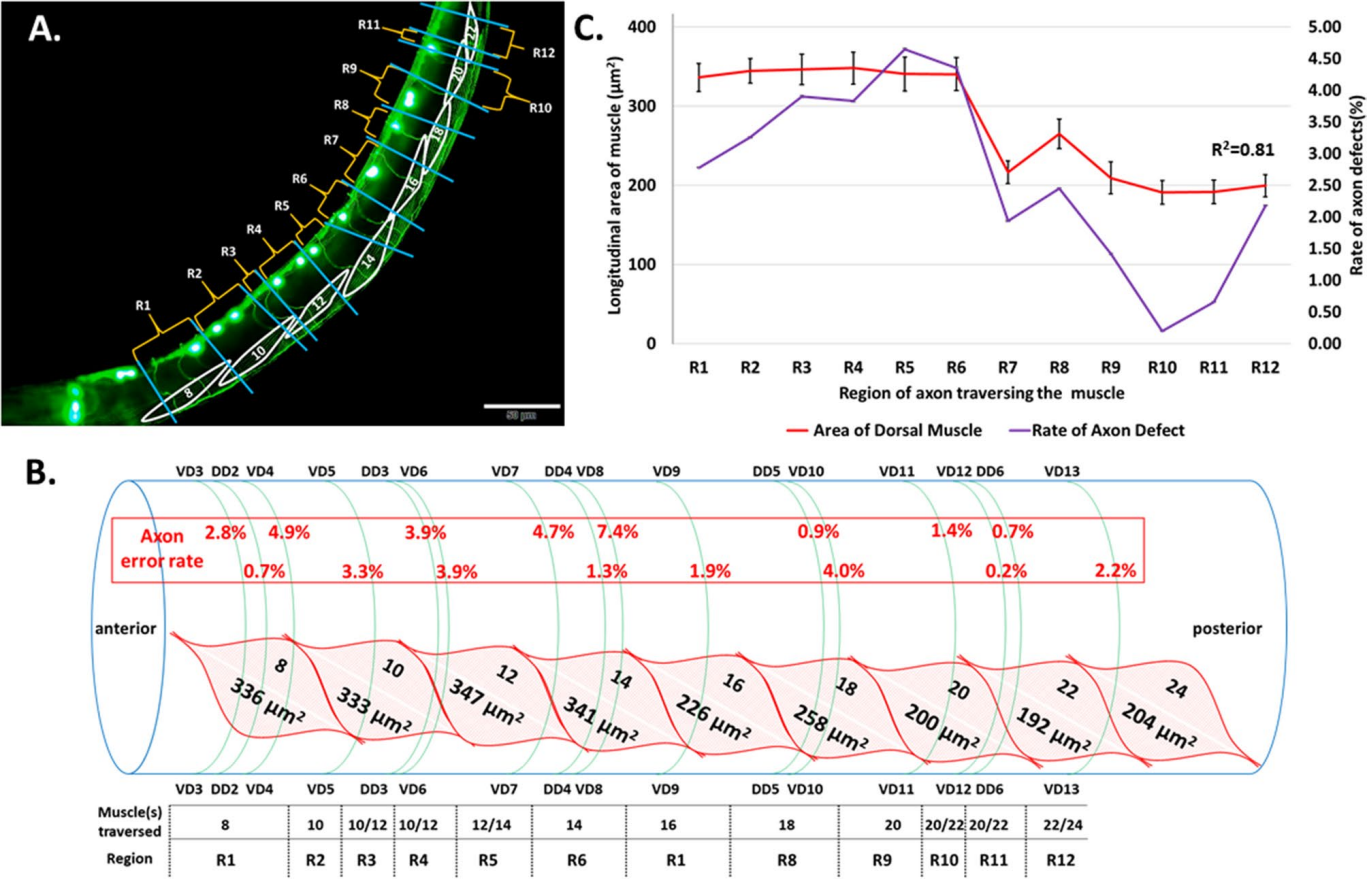
The frequency of DD/VD axon defects along the length of the animal is correlated with the size of the muscle that the axon traverses. (**A**) Dorsal lateral muscle cells (white) are labeled according to their numerical identity^20^. The regions R1 to R12, labeled in orange, are determined by the location of the muscle cell or cells that the axon traverses, and boundaries of each region are labeled in blue. Scale bars indicate 50 μm. (**B**) Model of *C. elegans* with dorsal lateral muscle overlayed by DD/VD neuron axons. Dorsal lateral muscle is depicted in a layered fashion in order to fit all 9 muscles. Below each muscle identity (muscles 8 to 24) is the average longitudinal area of each muscle (see Fig S2). DD/VD commissural axon identities are indicated, and the axon error rate for each axon is displayed. Below each axon identity, the muscle(s) traversed by the axon is indicated, and the represented region R1 to R12 is shown. (**C**) The longitudinal area of muscle cells (left Y axis, red line) in each region from the anterior side (R1) to the posterior side (R12) are plotted together with the average rate of axon defects (right Y-axis, purple line) in each region. Correlation (R^2^) for the two sets of data was calculated by regression analysis as 0.81. Error bars indicate standard error.

The adult *C. elegans* contains 95 rhomboid-shaped body wall muscle cells arranged in four longitudinal bundles, two dorsally and two ventrally, across the long axis of the worm^19^. Each muscle bundle contains 23 or 24 muscle cells, arranged in a medial row and a lateral row of cells. By the time of hatching, 81 of these muscle cells are already formed, and 14 more develop by the L1 larval stage, well before most of the DD/VD axons begin to form^41,42^. 16 of the DD/VD axons traverse around the left dorsal lateral muscle in a stereotyped pattern and location, albeit with some variation. As shown on Fig. 4A, B, we identified each dorsal lateral muscle, previously classified as muscle 8 to muscle 24^20^, that the 16 DD/VD neurons traverse. We found that 10 of the DD/VD axons always traverse the same muscle. For instance, the axons of the anterior neurons VD3, DD2 and VD4 always cross muscle 8 (Fig. 4B). On the other hand, 6 of the axons traverse either one muscle or the neighboring muscle. This is observed in the DD3 axon which can traverse either muscle 10 or muscle 12 (Fig. 4B). Next, we measured the longitudinal area of the dorsal lateral muscle cells in normal 1G conditions and found large differences in the size of muscle cells. We found that the area of muscle cells varies from 191 µm^2^ in muscle 22–347 µm^2^ in muscle 12, an 82% increase in size (Fig. 4C; Fig S2).

We asked if there was a relationship between muscle cell size and the frequency of axon errors observed in normal 1G gravity conditions. In order to analyze this, we first divided the areas where axons traversed the muscle into 12 regions called R1–R12 (Fig. 4B). R1 is the anterior-most region where the neurons VD3, VD4 and DD2 traverse through (Fig. 4A, B). R2, R3, R4, R5, R7, R9, R10, R11, R12 represent the regions of muscle where VD5, DD3, VD6, VD7, VD9, VD11, VD12, DD6, and VD13 neurons pass respectively, and R6 and R8 regions represent muscle area where axons of DD4-VD8 and DD5-VD10 traverse (Fig. 4A, B). To determine the frequency of axon defects, we used previously published data from our group showing the rate of axon defects for each DD/VD axon^22^. In the regions in which multiple axons traverse through, we calculated the average rate of defects.

Muscle size tended to be thicker in the anterior half of the worm at around 340 µm^2^. Midway through the length of the worm, there was a precipitous dip in cell size, followed by a slight increase, then a decrease again at the posterior side. The rate of axon defects generally followed a similar pattern, but the correlation was especially evident along the middle of the worm: where there was a precipitous decline in the size of muscle, axon defects also decreased. When muscle size was bigger in the next region, there was a coincident increase in the rate of axon defects, which decreased again as the muscle size became smaller in the following region. From this, we saw a clear correlation (R^2^ = 0.81) between the two plotted curves. This indicates that the incidence of axon errors is correlated to the size of the muscle cell that the axon traverses on the dorsal side.

Taken together, our data show that dorsal lateral muscles may act as a physical barrier for axons to reach the dorsal nerve cord, and that natural errors found in normal gravity conditions in our previous study as well as others^43,44^ may be due in large part to size of the muscle cell at the location of crossover independent of gravity conditions.

### Hypergravity decreases the size of muscle cells while increasing the incidence of axon defects

**Figure 5:**
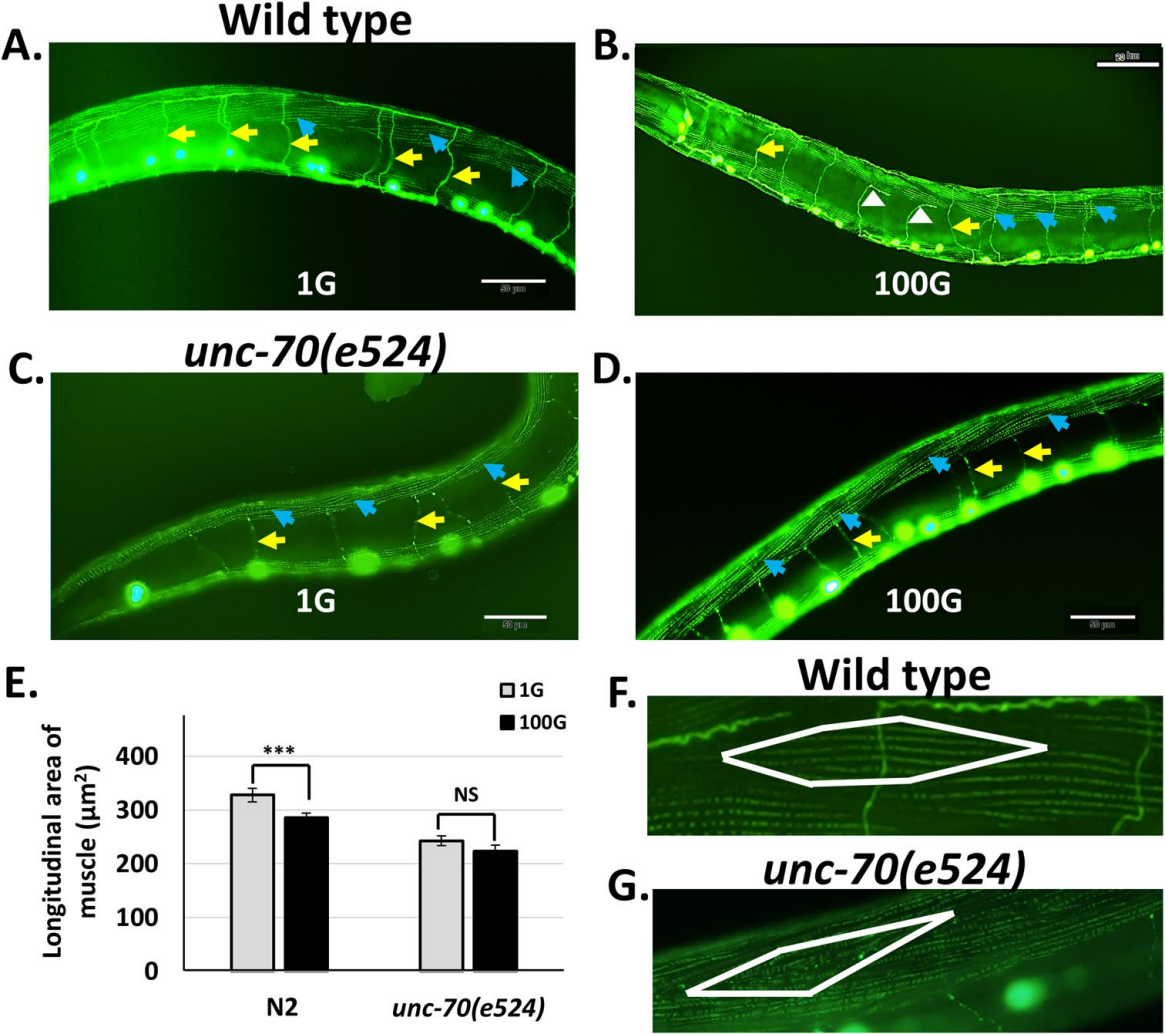
Hypergravity decreases muscle cell size. (A-D) 100G hypergravity in either wild-type N2 (**B**) or *unc-70* mutants (**D**) does not result in obvious changes in muscle structure when compared to normal 1G gravity in N2 (**A**) or *unc-70* (**C**). Yellow arrows indicate DD/VD axons, blue arrows indicate muscle cells, white arrowheads indicate defective axons. Scale bars indicate 50 μm. (**E**) 100G hypergravity decreases muscle cell size in wild-type animals but not in *unc-70* mutants. Error bars indicate standard error. Statistical significance was analyzed by student’s T-test. ***p < 0.001. (**F**–**G**) *unc-70* mutants display smaller muscle cells and subtle defects in actin organization. A single muscle cell is shown inside the white.

Since muscle size correlates with axonal defects, we wondered whether HIAD occurred due to an increase in muscle size in hypergravity. However, worms grown in 100G actually had slightly decreased muscle cell size (Fig. 5A, B, E). In addition, labeling muscle fibers with fluorescent-conjugated phalloidin did not reveal any obvious changes in muscle cell shape or actin organization in hypergravity (Fig. 5A, B). We concluded that, while muscle cell size and shape are correlated with axon defects in the 1G condition, other factors may contribute to hypergravity-induced axon defects.

We also looked at the muscle of *unc-70* mutants to see if there were any associated changes. UNC-70 localizes to the membrane attachment sites of the sarcomere at the sarcolemma, and null mutants or RNAi of *unc-70* result in animals with compromised sarcomere and sarcolemma structure^35,36^. In the *unc-70(e524)* hypomorph, we saw only subtle defects in actin organization at 1G normal gravity (Fig. 5A–D, F, G), and observed a 12.7% decrease in muscle cell area in the mutants (Fig. 5E). In 100G hypergravity, *unc-70* mutants displayed subtle defects in actin organization similar to 1G (data not shown), but we did not observe a further decrease in muscle cell longitudinal area as observed in the wild-type animals in hypergravity. This suggests that the suppression of HIAD in *unc-70* mutants is not due to changes in muscle cell size.

### Genes important for muscle-epidermal connections promote HIAD

As previously mentioned, VD axons must pass through the narrow basal lamina between the muscle and epidermis towards the dorsal nerve cord^37^. The extracellular landscape that they travel through consists of dense fibrous organelles scattered throughout the basal lamina/ECM. FOs play a major role in transmitting tension between the body wall muscle and the cuticle during movement^38,39^. In the absence of a bony skeleton, these connections from inner to outer layers allow the muscle to control the undulating movement of the worm. Actin and myosin muscle filaments attach to proteins in the membrane that then associate with the FOs in the ECM (Fig. 7). UNC-70 essentially acts both in the epidermis and muscles to strengthen this connection on either end^35,40^. Since *unc-70* expression in both muscle and epidermis promotes HIAD (Fig. 2), we hypothesized that suppression of HIAD in *unc-70* mutants could be explained by loosening of these dense connections that may impede the pathway of axons.

**Figure 6:**
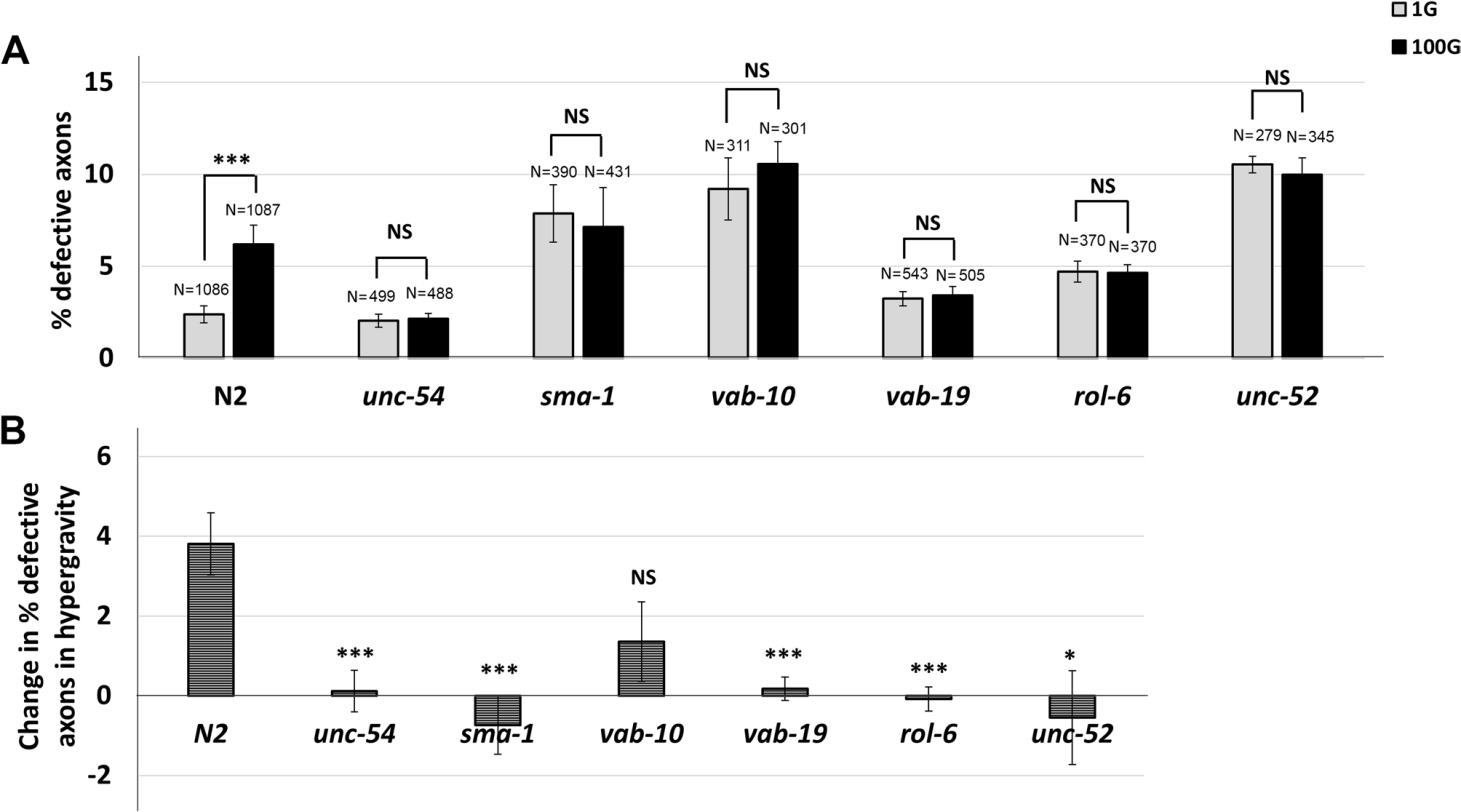
Mutants of genes that mediate the physical connection between muscle, epidermis and cuticle also promote HIAD. (**A**) Quantification of the percentage of axon defects observed at 1G and 100G for each strain. N = total number of animals analyzed. Error bars indicate standard error. Statistical significance between 1 and 100G for each strain was analyzed by student’s T-test. ***p < 0.001. NS indicates not significant. (**B**) Increase or decrease in the percent of defective axons in hypergravity. Error bars indicate standard error. Statistical significance between N2 and each mutant strain was analyzed by student’s T-test. *p < 0.05, ***p < 0.001, NS indicates not significant.

To test whether FOs are involved in inducing HIAD, we tested mutants of genes that are involved in promoting the structural integrity of FOs. Since FOs are a part of a network of connections originating from the muscle to the cuticle, we wondered whether defects in sarcomere organization in the muscle compromises these connections, suppressing HIAD. To test this hypothesis, we examined mutants of the *unc-54* gene, which encodes myosin heavy chain in skeletal muscles. Disruption of *unc-54* results in severe defects in muscle structure^45^. We found that HIAD was suppressed in *unc-54(s74)* mutants similar to *unc-70* mutants (Fig. 6, Fig S1, Fig S2).

Next, we tested *sma-1*, a β-H-spectrin expressed in the epidermis related to UNC-70 β-G-spectrin. SMA-1 is known to interact with components of fibrous organelles, presumably to attach FOs to the actin cytoskeleton of the epidermis^39,46^. Interestingly, *sma-1(ru18)* mutants also showed suppression of HIAD (Fig. 6, Fig S1, Fig S2).

**Figure 7:**
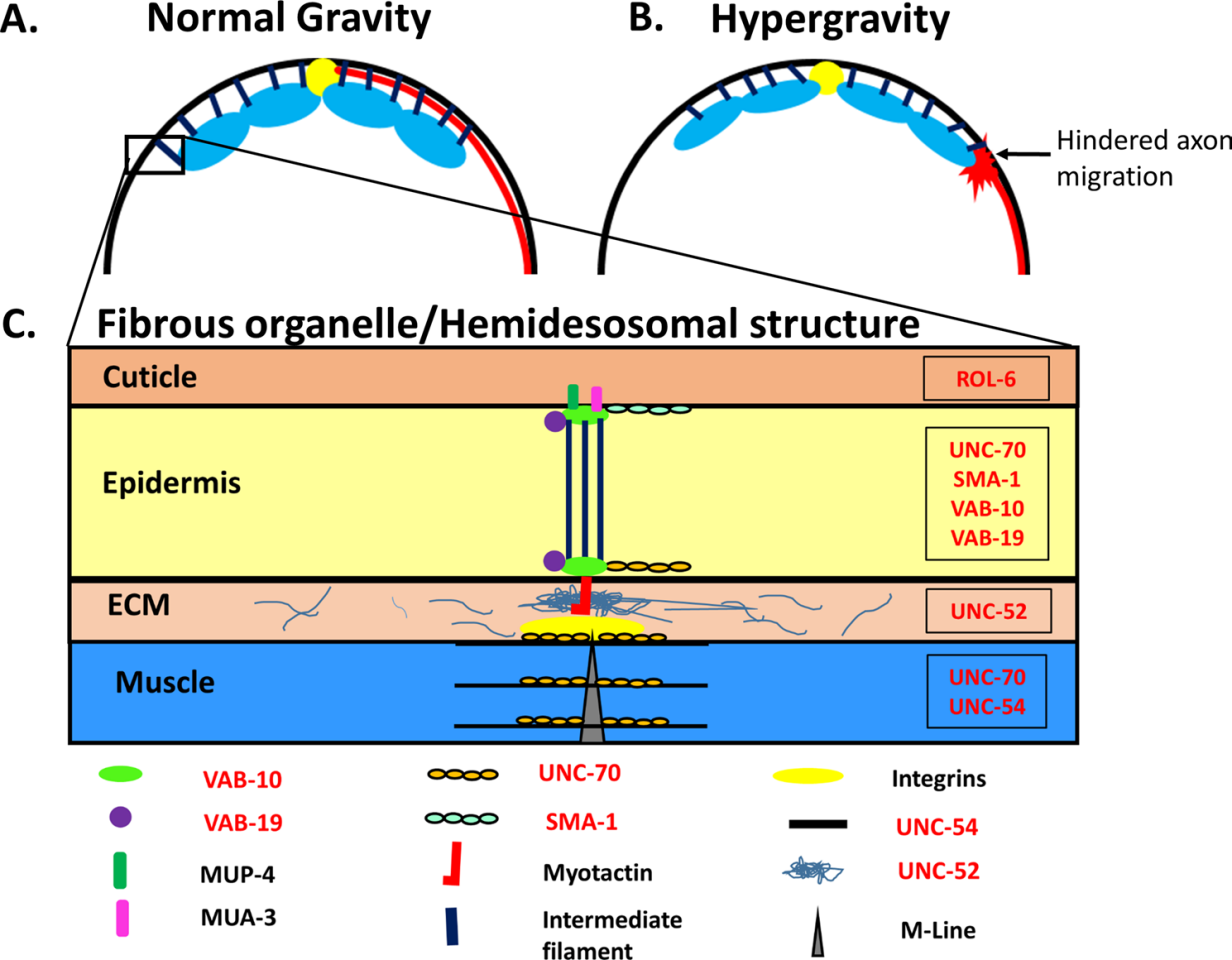
Putative model of hypergravity’s effect on tissue layers and hindered DD/VD axon migration. (**A**) Model for axon migration in normal gravity. The narrow ECM between muscle (blue) and epidermis is sufficient for growth cone passage, and fibrous organelle (black lines) density is normal allowing axon migration (red line) to occur normally. (**B**) Model for hindered axon migration in hypergravity conditions. ECM between muscle and epidermis is compressed, and FO density is increased, resulting in blocked axon migration (red star). (**C**) Magnified model of a single fibrous organelle shown in the inset in (**A**). Model of connections between the Muscle, ECM, epidermis and cuticle indicated, with the structure of an FO connecting the tissue layers shown in the middle. Proteins associated with the FO such as VAB-10, VAB-19, SMA-1 and UNC-70 in the epidermis and UNC-52 in the ECM associate with structures in the muscle that include UNC-54 and UNC-70 proteins. Proteins associated with the fibrous organelle are indicated below the model with those that were tested by genetic analysis in Fig. 6 indicated in red. On the apical side of the epidermis, these structures interact with collagen proteins that form the cuticle including ROL-6.

In addition to *sma-1,* we investigated the role of other proteins important to FOs and muscle-epidermis-cuticle connections. VAB-10 encodes a spectraplakin protein, and the VAB-10A isoform plectin protein associates with FO structures at the apical and basal sides of epidermal cells physically holding muscle, epidermal and cuticular tissues together during muscle contraction forces^47,48^. VAB-19, the ortholog of ankyrin repeat protein KANK1 expressed in the epidermis, is also associated with FOs through VAB-10^46^. UNC-52/Perlecan is a protein localized in the ECM between muscle and epidermis^49,50^, critically linking the two tissues through FOs (Fig. 7). ROL-6 is a collagen protein found in the cuticle. It is expressed in the epidermis, secreted on the apical side and crucial to the proper formation of the collagen-based cuticle^51^. Consistent with our hypothesis, hypergravity could not induce axon defects in *vab-10(e698)*, *vab-19(e1036)*, *unc-52(e1421),* and *rol-6(su1006)* mutants (Fig. 6A), could not increase the proportion of worms with axons of defects (Fig S2), and was significantly different compared to N2 wild type for 3 of the 4 mutant strains (Fig. 6B). Thus, disruption of the muscle-epidermis-cuticle connections allows DD/VD axon pathfinding in hypergravity. Taken together, we find that structural proteins that normally hold muscle, epidermal and cuticle tissue layers together can hinder axon migration in an altered gravity environment.

## Discussion

### A model for how hypergravity force promotes DD/VD motor neuron axon defects

Previously, we showed that hypergravity exposure increases DD/VD motor neuron axon defects^22^. Here, we sought to explain how gravity force can alter the axon development in the worm. Using a candidate genetic screen approach, we identified genes that can suppress hypergravity-induced axon defects. Among these, we found genes involved in tissue structure organization including the cytoskeletal protein UNC-70 β-spectrin. We showed by genetic rescue that UNC-70 expression in both muscle and epidermis is required for HIAD. Moreover, we showed that in addition to *unc-70*, structural proteins in the epidermis, ECM and cuticle that contribute to the structure and integrity of FOs were also required for HIAD.

We propose a model in which structural proteins that hold muscle-epidermal-cuticular tissue layers into proper positions may impede axon passage upon acute gravity force due to compression of tissue layers including the narrow basal lamina-ECM that the axons must migrate through (Fig. 7). We speculate that the disturbance of proteins that allow the muscle, epidermis and cuticle to remain physically connected loosens the rigid organization of the tissue layers allowing the passage of axons even in the presence of a strong hypergravity force. Further analysis of the role of myotactin and integrins, which are associated with FOs in the ECM^41,52,53^ and MUA-3, which is associated with FOs on the apical side of the epidermis^54^, should bolster support for this hypothesis.

How then does loss of these structural connectors allow DD/VD axons to easily reach their dorsal targets in the presence of hypergravity? The first consequence of this is the decreased density of structural proteins in the ECM. FOs densely line the ECM between muscle and epidermis. Loss of or decreased FOs would provide the axon growth cones a clearer path to their targets. The second consequence of decreased structural proteins is the increased space in the ECM. The ECM between muscle and epidermis is a narrow 20 nm space^55^. However, it has been shown that in mutants in which muscle separates from the epidermis, large pockets of space develop in the ECM^47,56^. This can potentially mitigate stalling and errors that normally occur in the tight ECM migration pathways. Further cell biological and ultrastructural analysis during DD/VD axon migration in the L1/L2 larval stages need to be conducted to confirm these hypotheses.

### Other factors that may influence HIAD

It is well established that axon outgrowth and guidance mechanisms allow the axon to reach proper targets, but we found that mutants of axon outgrowth and guidance as well as a mechanosensory mutant did not alter HIAD. This was not surprising given the fact hypergravity exposure for 18 h from embryo to early L1 was sufficient to induce axon defects^22^. Since VD neurons are born after the L1–L2 transition, hypergravity’s effect occurs before the neurons are even born. These two lines of evidence allowed us to negate the idea of a direct gravity effect on the neurons, and instead investigate other tissues or cell types.

In our previous characterization of DD/VD axon development, we found rare but consistent axon defects in wild-type animals under normal gravity conditions^22^. A noticeable pattern appeared in which defects occurred more frequently at certain axons than others. Here, we measured the longitudinal area of the dorsal muscle and found that the high-error rate axons generally traversed the larger muscle cells (Fig. 4). Since it is known that the physical presence of muscle causes the VD axons to stall at the dorsal lateral side^37^, and that the axon growth cone must navigate the narrow 20 nm basal lamina/ECM space between the muscle and epidermis^55^, we speculate that larger muscle could further narrow the basal lamina/ECM passageway impeding normal axon migration independent of gravity conditions.

In this line of reasoning, one may speculate that hypergravity increases muscle cell size to hinder axon migration. Contrary to this, hypergravity decreases longitudinal muscle size (Fig. 5). A previous report demonstrated that 100G hypergravity does not alter muscle structure but may increase sarcomere length slightly^16^. Since we have only measured the longitudinal size of the middle of the muscle rather than 3D muscle volume, we cannot confirm whether muscle cells are actually smaller in hypergravity. Further experiments will need to confirm the effect of hypergravity on actual muscle cell size.

We show that hypergravity does not increase DD/VD axon defects in mutants of *unc-*70. However, *unc-70* mutants themselves show slight increases albeit relatively low numbers of axon defects, and transgenic expression of *unc-70* also induced low levels of axon defects (Fig. 2). From previous studies we know that the loss of *unc-70* results in (1) DD/VD neuron axon fragility^29^; and (2) abnormal FO localization resulting in the loss of PLM neuron axon integrity in the epidermis^40^. Due to these roles of UNC-70, we surmise that altering levels of UNC-70 in the neurons, epidermis and muscle could result in increased DD/VD axon defects which appears to be separate from the increases observed in hypergravity in normal animals and the suppression observed in *unc-70* mutants.

We showed that mutations in genes expressed in epidermal cells including *unc-70* and *sma-1* β-spectrin genes and *vab-19* (Fig. 6) suppressed HIAD. Although their encoded protein are known to associate with FO structures, *sma-1* and *vab-19* also play important roles in epidermal elongation that allow the worm body to grow in size^46,57,58^, whereas a role for *unc-70* in body growth is unknown. During larval development, structures such as the DD/VD axon adjust themselves to the growing body by some unknown mechanism. An alternative explanation of why *unc-70* suppresses HIAD could be that hypergravity disrupts this mechanism during the L1 larval stage resulting in HIAD, and compensatory mutations that alter body growth such as *sma-1* and *vab-19* suppress HIAD. To assess whether *sma-1*, *vab-19,* and *unc-70* are all necessary for body elongation from L1 to L2 larval stages, we measured their body lengths two hours and again 18 h after hatching. However, *unc-70* mutants were normal for body length during early larval development (Fig S4). Thus, we do not observe any relationship between body elongation and HIAD.

Several mutants that suppress HIAD such as *unc-70* and *unc-54* are known to be important for proper muscle structure and development^35,36,59^. Indeed, we demonstrate that a weak of allele of *unc-70*^30^ shows smaller body wall muscle size than normal (Fig. 5). However, other mutants that suppress HIAD have normal muscle structure and development^46,51^. Further genetic analysis is needed to determine the relationship between muscle structure and HIAD. Another possibility that may explain genetic suppression of HIAD could be that hypergravity increases the FO density by increasing the number of FO structures in the ECM. However, additional analysis of FO protein expression and localization is required to ascertain this possibility.

### Application in vertebrates and mammals under hypergravity and microgravity

In this study we have chosen *C. elegans* as a model animal to investigate the effects of hypergravity on proper neuron development due to its ease of experimental use and availability of *C. elegans* genetic tools. As the ability of an animal to withstand gravity force is inversely correlated to its size and weight^60^, we sought to find a gravity force appropriate for *C. elegans*. Although mammals can withstand forces of 10-15G, *C. elegans* can withstand forces upwards of 400,000 G^61^. Since 100G hypergravity has been used in several previous studies^16,17,22^, we decided that this gravity force would be appropriate for our study and comparable to hypergravity forces experienced in higher animals during spaceflight.

We have demonstrated that in *C. elegans* hypergravity may be compressing rigid tissue layers together squeezing down on the sandwiched ECM space and indirectly causing defects in axons that migrate through the narrow channels of the ECM. Though the induced axon defects are significant and reproducible, subtle defects as those shown here and previously do not result in any noticeable change in locomotory behavior^62^. Our genetic experiments show that loss of proteins that promote cell and tissue adhesion can suppress the neuronal defects. Such genetic and tissue level hypergravity and microgravity experiments are possible in an invertebrate animal like nematodes but may pose a challenge in higher animals. However, clues that may support our hypothesis have been observed previously in mammalian studies. First, slowing of development and decreases in brain cell number or brain volume correspond with hypergravity exposure in the vertebrate nervous system^6–10^ including changes in the development of vestibular neuron axons^11^. Second, expression levels of the cell adhesion proteins CD15 and NCAM-L1 are associated with hypergravity-induced changes in the brain^9,12^. It would be interesting to explore whether hypergravity forces influence cell or tissue adhesion in vertebrates, and whether there are causative relationships between cell and tissue adhesion and hypergravity-induced changes observed in the vertebrate nervous system.

Furthermore, changes observed in laboratory-simulated or real space microgravity may be consistent with our observations. For instance hindlimb unloading experiments in mice resulted in decreases in expression of type IV collagen gene and the UNC-70 homologue perlecan gene in spinal cord^63^. Space-flown human T lymphocyte cells showed increased expression in the VAB-10-related human plectin gene and the UNC-54-related myosin gene^64^. Whether these gene expression changes are reflective of changes in cell or tissue interactions in response to microgravity environments need to further evaluated.

## Supplementary information

Supplementary Legends.

Supplementary Figure S1.

Supplementary Figure S2.

Supplementary Figure S3.

Supplementary Figure S4.
