# Supplementary material for: Muscle and epidermal contributions of the structural protein β-spectrin promote hypergravity-induced motor neuron axon defects in C. elegans
Source: Sci Rep. 2020 Dec 3;10:21214. doi: 10.1038/s41598-020-78414-y (PMC7713079; doi:10.1038/s41598-020-78414-y)
Supplement: Supplementary file 1 — Supplementary Legends. [file 41598_2020_78414_MOESM1_ESM.docx]

**Muscle and epidermal contributions of the structural protein β-spectrin promote hypergravity-induced motor neuron axon defects in *C. elegans***

**Running title: Hypergravity causes axon defects through epidermal and muscle β-spectrin**

Saraswathi Subbammal Kalichamy^1^, Alfredo V. Alcantara Jr.^1^, Ban-Seok Kim^1^, Junsoo Park^1^, Kyoung-hye Yoon^2^ and Jin I. Lee^1^

^1^ Division of Biological Science and Technology, College of Science and Technology, Yonsei University, Mirae Campus, Gangwon-do, South Korea

^2^ Department of Physiology, Mitohormesis Research Center, Yonsei University Wonju College of Medicine, Wonju, Gangwon-do, South Korea

^*^ Corresponding author:

Jin I. Lee: Mirae 304, 1 Yonseidae-gil, Wonju, Gangwon-do 26493, South Korea, [jinillee@yonsei.ac.kr](mailto:jinillee@yonsei.ac.kr), tel: +82-33-760-2249, fax: +82-33-760-2183

**Supplemental Figure Legends**

Figure S1. Candidate genetic screen for genes that regulate HIAD. Each data point connected by dotted lines represents one paired trial. Total number of animals counted is indicated in Figure 1i. Bars representing average percent axon defects is the same as shown in Fig 1 and Fig 6. *mig-15* and *unc-129* mutants are shown on the right graph separately due to a much larger baseline axon defects in these two strains.

Figure S2. Proportion of animals with at least one DD/VD axon defect. 100% of *unc-129* mutant animals had at least one axon defects. Error bars indicate standard error. Statistical significance was determined by student’s T-test. * indicates p<0.05, ** indicates p<0.01, *** indicates p<0.001, NS indicates no significance.

Figure S3. Cross sectional area of individual dorsal lateral muscles with standard error.

Figure S4. Mutants of *unc-70* display normal growth through the L1 larval stage. *sma-1* mutants are known to have small body length, whereas *lon-2* mutants are known to have long body length. *vab-19* is known to be involved in elongation, and *vab-19* mutants display long body length through the L1 larval stage compared to wild-type N2. Error bars indicate standard error. Statistical significance was determined by student’s T-test. NS indicates no significance. *** indicates p<0.001.
