# Supplementary figures and images for: Muscle and epidermal contributions of the structural protein β-spectrin promote hypergravity-induced motor neuron axon defects in C. elegans
Source: Sci Rep. 2020 Dec 3;10:21214. doi: 10.1038/s41598-020-78414-y (PMC7713079; doi:10.1038/s41598-020-78414-y)

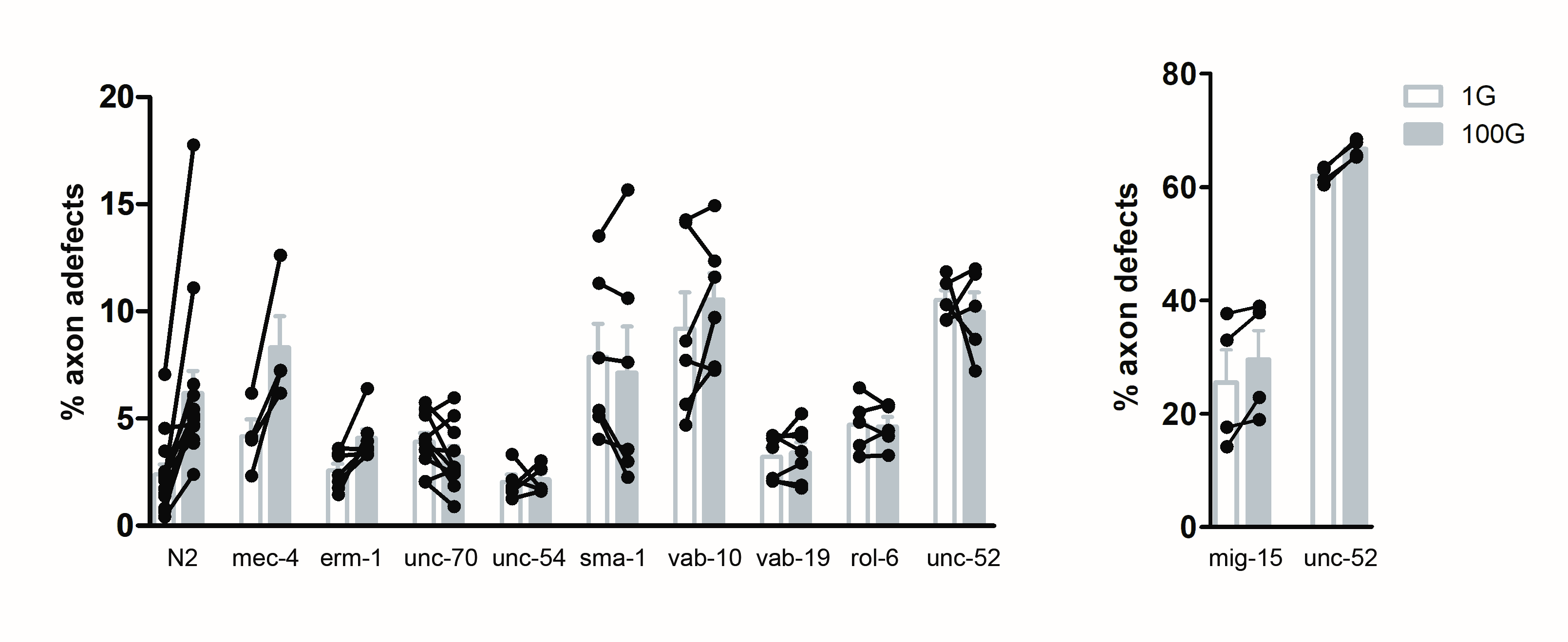

Supplement: Supplementary file 2 — Supplementary Figure S1. [file 41598_2020_78414_MOESM2_ESM.tif]

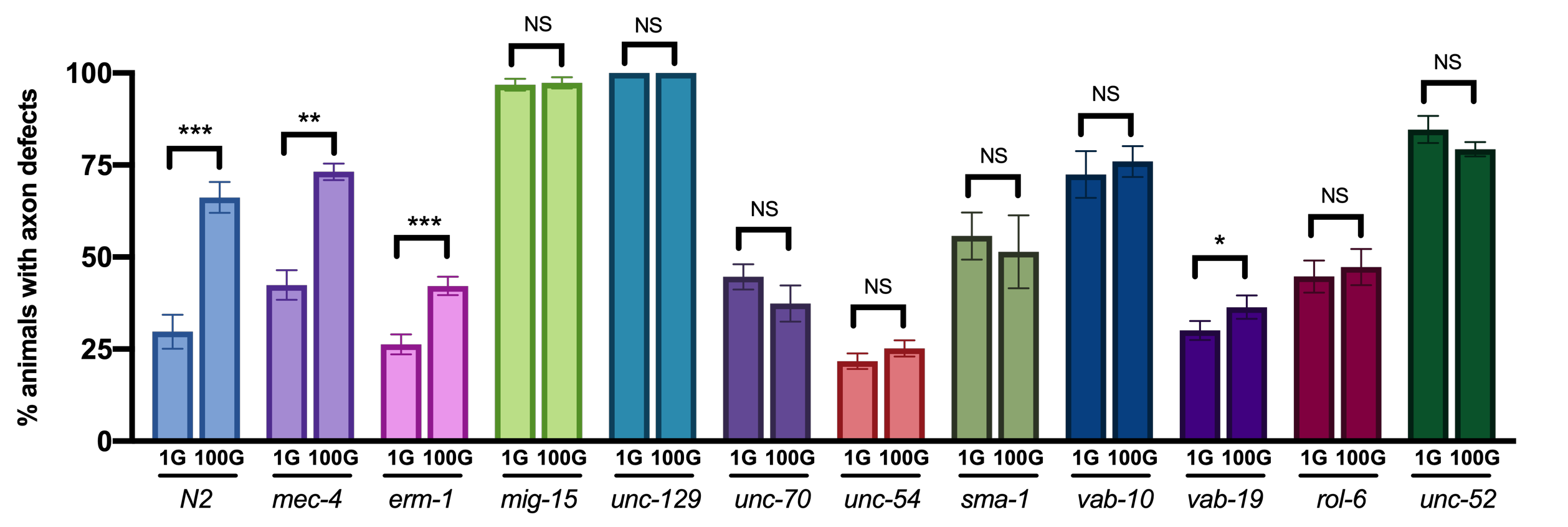

Supplement: Supplementary file 3 — Supplementary Figure S2. [file 41598_2020_78414_MOESM3_ESM.tiff]

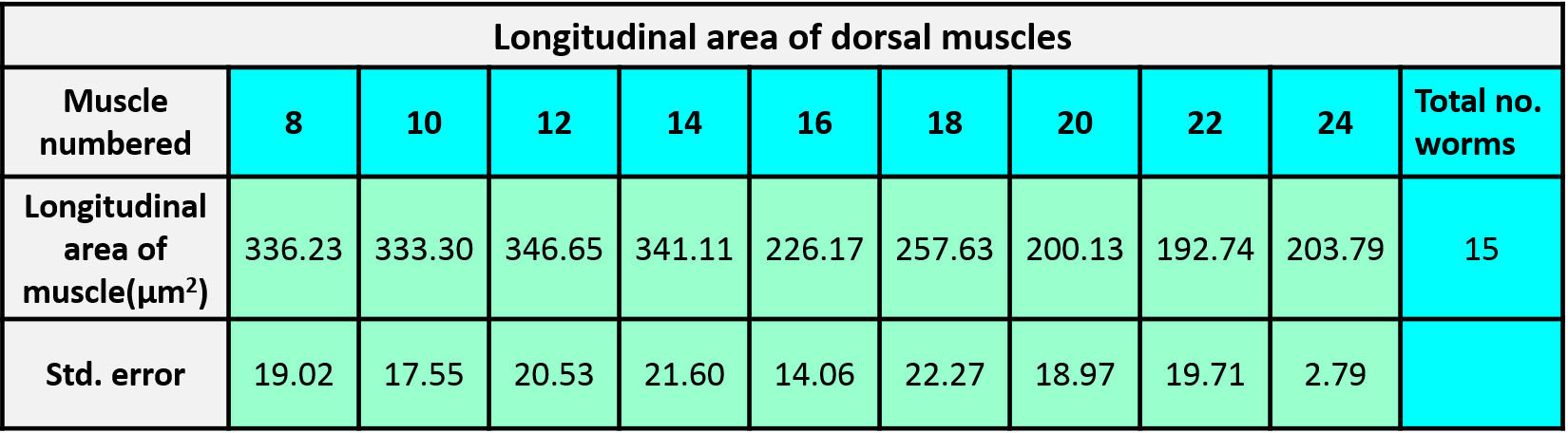

Supplement: Supplementary file 4 — Supplementary Figure S3. [file 41598_2020_78414_MOESM4_ESM.tif]

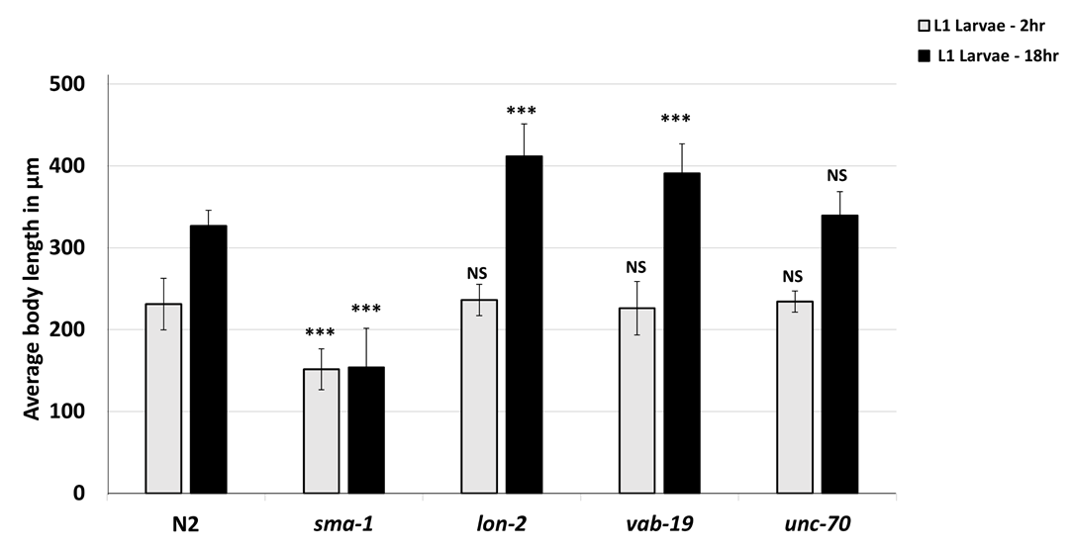

Supplement: Supplementary file 5 — Supplementary Figure S4. [file 41598_2020_78414_MOESM5_ESM.tif]
